# Directional telomeric silencing and lack of canonical *B1* elements in two silencer Autonomously Replicating Sequences in *S. cerevisiae*

**DOI:** 10.1186/1471-2199-13-34

**Published:** 2012-11-16

**Authors:** Patricia Chisamore-Robert, Samantha Peeters, Kristina Shostak, Krassimir Yankulov

**Affiliations:** 1 Department of Molecular and Cellular Biology, University of Guelph, Guelph, ON, Canada; 2 Current address: Department of Medical Genetics, Molecular Epigenetics Group, University of British Columbia, Vancouver, Canada

**Keywords:** Autonomously replicating sequences, Telomere position effect, DNA replication, Gene silencing

## Abstract

**Background:**

Autonomously Replicating Sequences (*ARS*) in *S. cerevisiae* serve as origins of DNA replication or as components of *cis*-acting silencers, which impose positional repression at the mating type loci and at the telomeres. Both types of *ARS* can act as replicators or silencers, however it is not clear how these quite diverse functions are executed. It is believed that all *ARS* contain a core module of an essential *ARS* Consensus Sequence (*ACS*) and a non-essential *B1* element.

**Results:**

We have tested how the *B1* elements contribute to the silencer and replicator function of *ARS*. We report that the *ACS-B1* orientation of *ARS* has a profound effect on the levels of gene silencing at telomeres. We also report that the destruction of the canonical *B1* elements in two silencer *ARS* (*ARS317* and *ARS319*) has no effect on their silencer and replicator activity.

**Conclusions:**

The observed orientation effects on gene silencing suggest that *ARSs* can act as both proto-silencers and as insulator elements. In addition, the lack of *B1* suggests that the *ACS-B1* module could be different in silencer and replicator *ARS*.

## Background

Origins of DNA replication in budding yeast are well defined DNA elements referred to as Autonomously Replicating Sequences (*ARS*). They consist of a core 11 base pair *ACS* (*ARS* Consensus Sequence, WTTTAYRTTTW) and three or four auxiliary *B* elements [1]. *ACS* is critical for the function of *ARS*[2-4]. It is the main site of binding of the Origin Recognition Complex (ORC), which nucleates the formation of pre-replicative complexes in G1 [5,6]. It has been shown that the flanking sequences of the core *ACS* can also contribute to the binding of ORC thus producing the 17 base pair extended *ACS* (*EACS*) [7,8]. Compared to *ACS*, the *B* elements are not so well characterized and their roles are poorly understood. *B2* is believed to act as a site of DNA unwinding that allows for the initiation of replication [9,10]. *B3* is a binding site for Abf1p [11]. While the significance of these elements for the activity of the origins is apparent, the mechanism of their action is unclear. The *B1* element is positioned about 15 bases upstream of the core *ACS*. Using *ARS1* as a model, it has been shown that *B1* acts as a second binding site for ORC [5,6]. Earlier studies have proposed an AWnY consensus 14 bases upstream of *ACS*[12]. A more comprehensive analysis and alignment of multiple origins has shown better agreement for a WTW motif positioned 15–17 bases upstream of the core *ACS*[8]. Mutations in these WTW motifs have substantially reduced the replicator activity of most of the tested origins [8,13]. It has been proposed that the *B1* element together with the extended *ACS* produce a variety of bi-partite sites that bind ORC with different affinities [7,12,14].

Besides their role in DNA replication, *ARS* and ORC play a central role in gene silencing at the constitutively repressed mating type loci *HML* and *HMR* and in the subtelomeric regions of the chromosomes [1]. The silencers, which flank *HML* and *HMR* and impose complete shut-off of the genes between them, are built up of various combinations of binding sites for Abf1p and Rap1p plus one of four *ARSs (ARS301, ARS302, ARS317, ARS318) *[1]. Mutations in the *ACS* of these *ARSs* substantially reduce gene repression and confer inability to mate [1]. Interestingly, at the *HML-I* and *HMR-E* silencers the orientation of the *ACS-B1* elements of the *ARSs* has a directional effect on the levels of gene repression [15-17]. Multiple *ARSs* are also found in the *core X* and *Y’* subtelomeric regions [18,19]. At these positions they act as proto-silencers meaning that they relay and enhance the repression signals emitted by the telomeres [20]. It is not known if these *ARSs* have directional function. The *ARSs* at the subtelomeric and the mating type loci rarely fire at their native locations thus strengthening the notion that they have a silencing function independent of the initiation of DNA replication. However, when moved to a mini-chromosome or at different genomic position, these *ARSs* can fire as efficiently as any other *ARS*[21,22]. The basis of this dual function of *ARSs* is not fully understood [21].

Recent studies have shown that targeted mutations in the putative *B1* (WTW) motifs of certain *ARSs* have little effect on their replicator activity [4,8]. It is unclear if the same mutations affect gene silencing. It remains possible that such *ARSs* contain a *B1* at a different position. Ultimately, it is unclear if *B1* plays a role in *ARS*-dependent gene silencing. In this study we have tested the role of *B1* by parallel silencing and replicator assays. We have found that the putative *B1* elements of two silencer *ARSs* (*ARS317* and *ARS319*) are dispensable for both activities.

## Results

### Experimental strategy

**Figure 1:**
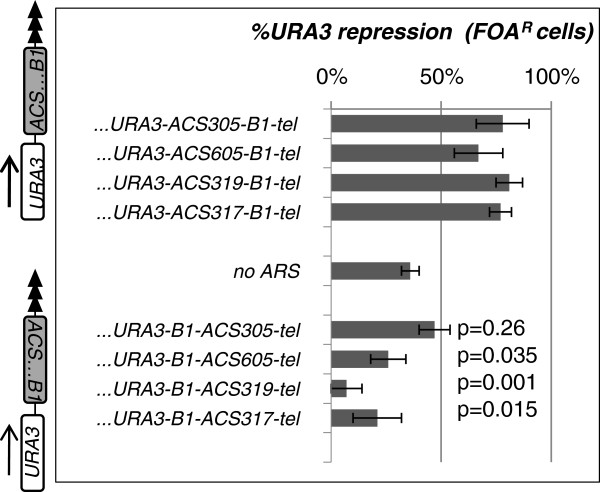
**Orientation-dependent silencing by *ARSs*. ***ARS305, ARS605, ARS317* and *ARS319* were cloned in *URA3-ACS-B1-tel* (upper part) and *URA3-B1-ACS-tel* (lower part) orientation and inserted in the *VIIL* telomere. Levels of *URA3* silencing were assessed as % FOA^R^ cells and plotted. Data is from Table 2. Statistical significance (*p* values) for the difference between the control construct (no *ARS*, middle of the graph) and the *ARSs* in *URA3-B1-ACS-tel* orientation are shown next to each bar. The *p* values for the constructs in *URA3-ACS-B1-tel* orientation are significantly lower than 0.05 and are not shown.

**Figure 2:**
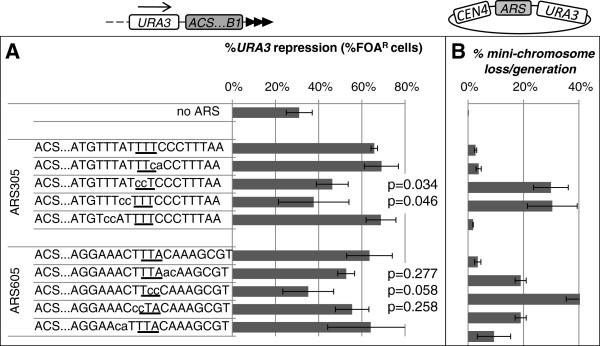
**Proto-silencer and replicator activity of *ARS305 *and *ARS605*. A**. The *ARSs* and their mutant derivatives (shown on the left) were integrated in the *VIIL* telomere in *URA3-ACS-B1-tel* orientation. The WTW motifs are underlined. The mutations are depicted by small letters. The silencer activity of the constructs was assessed as % FOA^R^ cells and plotted. Data is from Table 1. Statistical significance (*p* values) of the difference between the non-mutated construct and some of the mutants are shown next to each bar. The *p* values for the remaining constructs are significantly above 0.05 and are not shown. **B**. The *ARSs* and their mutant derivatives were cloned between *CEN4* and *URA3* to produce mini-chromosomes as indicated on the top of the figure. All mini-chromosomes were transformed in *W303* cells and loss per generation was calculated. Data is from Table 1.

**Figure 3:**
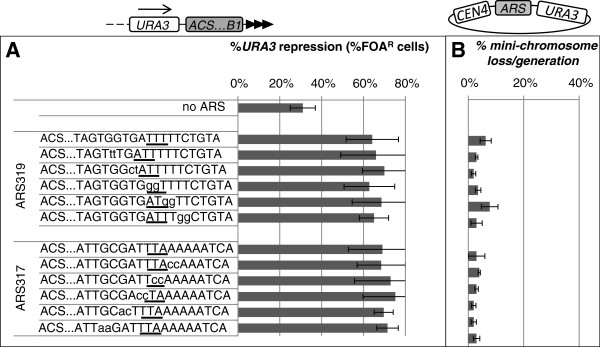
**Proto-silencer and replicator activity of *ARS317 *and *ARS319*. A**. The *ARSs* and their mutant derivatives (shown on the left) were integrated in the *VIIL* telomere in *URA3-ACS-B1-tel* direction. The putative WTW motifs are underlined. The mutations are depicted by small letters. The silencer activity of the constructs was assessed as % FOA^R^ cells and plotted. Data is from Table 1. The *p* values for all constructs are above 0.05 and are not shown. **B**. The *ARSs* and their mutant derivatives were cloned between *CEN4* and *URA3* to produce mini-chromosomes. All mini-chromosomes were transformed in *W303* cells and loss per generation was calculated and plotted. Data is from Table 1.

To address the role of *B1* in gene silencing, we isolated two silencer *ARS* (*ARS317* from the mating type *HMR* locus and *ARS319* from the *IIIR* subtelomeric region) and two well-characterized replicator *ARS* (*ARS305* and *ARS605*). These were inserted next to the *VIIL* telomere in *ACS-B1* and *B1-ACS* orientation (Figure 1) and the silencing of the adjacent *URA3* was assessed. In addition, we performed scanning mutagenesis of the *B1* elements of these *ARSs* and estimated the effects of the mutations on their silencer and replicator activity (Figure 2, Figure 3).

The silencer activity of all the *ARSs* was assessed by a routine TPE (Telomere Position Effect) assay [23]. The rationale of the assay is as follows: Telomeres recruit multiple Rap1 proteins, which in turn recruit Sir2/3/4 proteins [24,25]. The Sir proteins then spread over and de-acetylate the neighboring nucleosomes to establish a heterochromatin domain [1]. Depending on the scope of spreading, subtelomeric genes are either active or completely repressed and infrequently switch between the two states [23]. When *URA3* is inserted at the *VIIL* telomere (or at any other telomere) the proportion of repressed *URA3* within a cell population is modulated by the strength of the subtelomeric proto-silencer elements [20]. This proportion is easily assessed as %FOA resistant cells ((FOA (5-fluoro-orotic acid) is converted to a toxin by the enzyme encoded by *URA3))*[23]. Hence, in our assays the *per cent* FOA-resistant cells represents the proto-silencer strength of the engineered *ARSs*.

Parallel assays were conducted to test how the mutations in the *B1* elements affect the replicator activity of the four *ARSs* and if these effects correlate to the decrease in *ARS*-driven silencing (Figure 3). To this end, all wild type and mutant *ARS* fragments were sub-cloned in mini-chromosomes containing *CEN4* and *URA3* (Figure 3) and mini-chromosome stability assays were performed. *ARS/CEN* mini-chromosomes are replicated once per cell cycle and are properly segregated during mitosis. Their normal loss rate is about 3-5% per generation [22,26]. In our analyses any increase in the loss rate is indicative of malfunctioning of the origin. This assay provides a highly sensitive measure of the activity of the origins and has been instrumental in the deciphering of the regulatory elements in many *ARS’s*.

### The orientation of *ARS*s determines the level of telomeric silencing

It has previously been shown that *ARS317* exerts orientation-dependent silencing when the whole *HMR-E* silencer is moved to the *HML* locus [16]. The possibility of directional silencing prompted us to establish an orientation in which all analysed *ARSs* would produce similar levels of *URA3* silencing. *ARS305*, *ARS605, ARS317* and *ARS319* were sub-cloned in both directions (*ADH4URA3-ACS-B1-tel* and *ADH4-URA3-B1-ACS-tel*) and then inserted between *ADH4* and the *VIIL* telomere of *W303* cells as described previously [4,27,28]. Consistent with their established role as proto-silencers [20], in the *URA3ACS-B1-tel* orientation all *ARSs* increased the proportion of FOA resistant (FOA^R^) cells to 67-81% relative to 36% in the construct with no *ARS* (Figure 1, upper part). No significant difference between silencer and replicator *ARS* was observed. In the opposite *URA3B1-ACS-tel* orientation *ARS305* produced FOA^R^ values comparable to the construct without any *ARS* (Figure 1, lower part). Remarkably, *ARS317, ARS605* and especially *ARS319* produced statistically significant decrease (*p < 0.05*) of silencing relative to the control thus clearly displaying anti-silencing properties. In summary, the orientation of *ARS* relative to the telomere has a major impact on the level of subtelomeric silencing. From a technical point of view, in the *URA3ACS-B1-tel* orientation all *ARSs* showed similar proportions of FOA^R^ cells that allow for direct comparison of the role of their *B1* elements in gene silencing.

### The same *B1* elements in *ARS305* and *ARS605* contribute to gene silencing and DNA replication

Next, we measured the contribution of the *B1* elements to DNA replication and to gene silencing. We mutagenized the replicators (*ARS305* and *ARS605*) and silencers (*ARS317* and *ARS319)* in the region encompassing their putative *B1* (WTW) motifs by replacing two bases at a time (Figure 2A, Figure 3A). All wild type and mutant *ARSs* were cloned in *ADH4-URA3-ACS-B1-tel* orientation, inserted in the *VIIL* telomere and subjected to the FOA-resistance assays. In parallel, the same *ARSs* were cloned in *URA3/CEN4* mini-chromosomes and tested for their replicator activity. The substitutions in the WTW motifs (shown by the rectangle in Figure 2A) of *ARS305* and *ARS605* caused statistically significant decrease in the proportion of FOA^R^ cells relative to their non-mutated counterparts (Figure 2A). The flanking sequences in *ARS305* had little effect, while in *ARS605* they produced some minor reduction in silencer activity. In the mini-chromosome stability assay exactly the same mutations caused substantial increase of the loss per generation rates (Figure 2B). There is a good agreement in the magnitude of effects in the two assays with all mutants tested (Figure 2 A and B). These observations indicate that the same *B1* elements in *ARS305* and *ARS605* contribute to their silencer and replicator activity.

### Silencer *ARSs* lack functionally identifiable *B1*

Similar analyses of the two silencer *ARSs* (*ARS317* and *ARS319*) showed that none of the two-base substitutions in the vicinity of the WTW element altered the levels of *URA3* silencing (Figure 3A). To warrant for the existence of aberrantly positioned *B1* we expanded the scanning substitutions as compared to *ARS305* and *ARS605,* but no effects were observed. Similarly, the replicator activity of *ARS317* and *ARS319* remained largely unaffected by the mutations (Figure 3B). Only one of the *ARS319* constructs showed modest increase in the loss rate of the mini-chromosome, but the mutation was outside of the canonical WTW element. Clearly, both assays failed to reveal a *B1* element in *ARS317* and *ARS319.*

## Discussion

### Lack of canonical *B1* element

While the position and consensus of the *B2*, *B3* and *B4* auxiliary elements vary between different *ARSs*, the *ACS-B1* module serves as a binary binding site for the association of ORC and seems highly conserved [2,8,11,29,30]. Indeed, recent studies have identified a strong WTW consensus 15–17 bases downstream of the core *ACS*[8,13]. Mutations in this *B1* motif have caused significant loss of replicator activity in most *ARSs*[8,13], but its role in gene silencing has not been determined. Even more, mutations in the WTW consensus of one silencer *ARS* (*ARS317*) did not affect its replicator activity [8]. It seems conceivable that *ARSs* could utilize the WTW motif for replication and use an alternative *B1* element for gene silencing [21]. It also seems possible that silencer and replicator *ARSs* have different *B1* elements and different type of interaction with ORC [4,21]. Here we have addressed both possibilities. We have shown that exactly the same mutations impair the replicator and the silencing function of the tested replicator *ARSs* (Figure 2). Hence, the answer to the first question is negative: these two functions are not determined by alternative *B1* elements.

**Figure 4:**
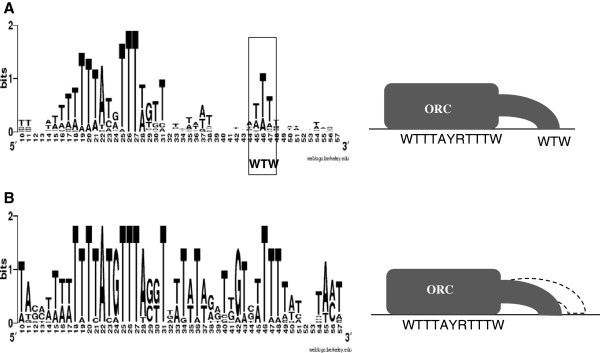
**Alignment of replicator and silencer *ARSs*.** The *ARSs* listed below were aligned using WebLogo *(weblogo.berkely.edu*). Diagram depicting the possible binding of ORC is shown on the right. **A**. Alignment of 25 replicator *ARS* as in [8]. The list of *ARSs* is available upon request. The WTW consensus of the *B1* element is shown in the rectangle. **B**. Alignment of subtelomeric *ARS*. *ARS319* was used to search for related sequences by BLAST. 144 matches were identified. The top 20 entries were all located in the subtelomeric regions. These were aligned at the *ACS* (WTTTAYRTTTW) and analyzed by WebLogo.

Surprisingly, the analyses of the two silencer *ARSs* (*ARS317* and *ARS319)* have revealed that neither the replicator nor the silencer function was affected by any of the two-base substitutions within the 10 base region of the putative *B1* (Figure 3). Again, we failed to obtain any evidence in favor of optional usage of *B1* in silencing and replication. However, it is apparent that *ARS317* and *ARS319* have a different *B1* or do not possess one at all. In the *core X* and *Y’* subtelomeric elements there are more than 100 close matches to *ARS319* with high levels of homology in their *ACS-B1* module (Figure 4B). We suggest that all these plus *ARS317* represent a subclass of *ARS* with a novel type of *B1* element or with no *B1* element.

*ARS* display considerable diversity of the sequences surrounding the core *ACS*[8,13,31]. To this diversity we add the extreme case of lack of a canonical *B1*. This is an intriguing issue. Being a secondary site for ORC binding, *B1* is expected to be important if not essential. It is possible that we have identified no *B1* element because in silencer *ARS* there is no second site of association for ORC. For example, a specialized extended 17 base pair *EACS*[7,8,12] could provide a single high-affinity site for the binding of ORC and minimize the significance of the *B1* element. Alternatively, these *ACS-B1* sites are bi-partite, but *B1* is broader. If this is the case, the two-base substitutions that normally destroy the WTW in replicator *ARSs* will not work on *ARS319* and *ARS317*. Indeed, the alignment of *ARS319* and other telomeric *ARS*s shows more than one potential WTW site in the area of *B1* (Figure 4B). The same applies to *ARS317* (not shown). However, the two *ARS* from the *HML* mating type locus do not show significant similarity to the putative *B1* regions of *ARS319* and *ARS317* (not shown). Even more, the high overall homology of sub-telomeric *ARSs* (Figure 4B) precludes the recognition of a different *B1* even if it existed. Hence, while a broader *B1* in silencer *ARS* remains possible, a consensus is difficult to identify. Ultimately, it is possible that a combination of an extended *ACS*[12,13,31] and broader *B1* provide alternatives for the binding of ORC that are hard to unveil by alignment algorithms.

Within eukaryotes, budding yeasts are the only known species with well defined sequences of origins and well defined binding sites for ORC. In all other species the origins are quite dissimilar and are recognizable only as A/T rich regions of DNA. This study together with other recent studies [8,12,13] suggests a certain level of diversity between different *ARS* in budding yeast. The significance of this diversity is yet to be determined.

### Directionality of silencing

Silencers and proto-silencer in *S. cerevisiae* are *cis*-elements that serve as focal points for the recruitment of Sir proteins [1] and in most cases are thought to act in bi-directional fashion. However, while selecting the optimal conditions for our silencing assays we came across strong and reproducible orientation-dependent effects. All *ARSs* were potent proto-silencers only in the *URA3-ACS-B1-tel* orientation, the same orientation found in natural sub-telomeric *ARS* elements. In the opposite *URA3-B1-ACS-tel* orientation *ARS319* displayed strong anti-silencing activity while *ARS317* and *ARS605* caused modest de-repression of *URA3*. These experiments have not been developed to establish if the *ACS-B1* module or other unknown sites within the cloned fragments exert these directional effects. However, several arguments support the idea that *ACS-B1* could play a central role. Earlier research has acquired evidence for directional silencing by *HMR-E* when it is inserted in the *HML* locus [15,17]. *HMR-E* consists of *ARS317* and binding sites for Abf1p and Rap1p. Gene repression has been robust at the Abf1p binding site of *HMR-E* and weak at the *ACS* side [15]. A stably positioned nucleosome was found adjacent to the *ACS* site of *ARS317* and not on the side containing the *B1* element [15]. The binding sites for Abf1p and Rap1p also contribute to the directionality of *HMR-E*. It is noteworthy that, excluding *ARS317*, none of our other constructs contains identifiable Abf1p or Rap1p binding sites thus leaving *ACS-B1* as a likely candidate for the effects we have observed. In addition, *ARS319* and not *ARS317* displayed the strongest directional effects (Figure 1). If *ACS-B1* is the key directional element, a stably positioned nucleosome next to *ACS* can stimulate the transfer of Sir proteins approaching from the telomere thus acting as a relay point. In the opposite orientation, a nucleosome-free DNA generated by the association of ORC [32] could prevent Sir protein spreading and act as a chromatin insulator [33]. However, why nucleosome-free DNA on one side of *ARS* would work as insulator in one orientation is yet to be established. Structural studies on ORC bound to different *ACS-B1* modules can address this possibility.

## Conclusions

All tested *ARS* display proto-silencing activity in the *ACS-B1*-*tel* orientation relative to the telomere. However, in the *B1-ACS-tel* orientation *ARS305* does not show proto-silencing activity, while *ARS605, ARS317, ARS319* display anti-silencing activity. Hence, there is a strong orientation dependency in the proto-silencing activity of *ARS*. In addition, *ARS317* and *ARS319* do not possess a canonical *B1* element thus suggesting a different *ACS-B1* module relative to *ARS305, ARS605* and numerous replicator *ARS*.

## Materials and methods

### Constructs

*ARS305, ARS605, ARS317* and *ARS319* were amplified by PCR from the genomic DNA of *W303* strain using Phusion polymerase (NEB) according to the instructions of the manufacturer. The genomic coordinates (as per the updates available in January 2010) of the amplified fragments are as follows: *ARS305, III:* 39392–39774; *ARS605, VI:* 135860–136202; *ARS317, III:* 292894–292369; *ARS319, III:* 315639–315989. The primer sequences are available upon request. The amplified fragments were sub-cloned in the BamH1 site of pUCAIV [27] between the telomeric TG_1-3_ repeats and the *URA3* reporter. Our constructs do not contain any additional sub-telomeric elements. Two bases at a time were replaced in the vicinity of the *B1* elements of the *ARSs* by site-directed mutagenesis. The mutated sequences are shown in Table 1. All mutations have been confirmed by DNA sequencing. The nucleotide sequences of the primers used to amplify genomic DNA and to mutate the cloned fragments are available upon request. 

**Table 1:** **Silencer and replicator activity of mutated *ARSs***

		**Silencer activity (FOA-resistance assay)**			**Replicator activity (mini-chromosome stability assay)**		
		**FOA**^**R**^		**STD**	**Loss/generation**		**STD**
	*no ARS*	31%	(n = 9)	6%	n/a		
*ARS305*	ACS…ATGTTTAT**TTT**CCCTTTAA	66%	(n = 9)	2%	3%	(n = 9)	0%
	ACS…ATGTTTAT**TTc**aCCTTTAA	69%	(n = 9)	8%	4%	(n = 9)	1%
	ACS…ATGTTTAT**ccT**CCCTTTAA	46%	(n = 9)	7%	30%	(n = 9)	6%
	ACS…ATGTTTcc**TTT**CCCTTTAA	38%	(n = 9)	16%	30%	(n = 9)	9%
	ACS…ATGTccAT**TTT**CCCTTTAA	69%	(n = 9)	7%	2%	(n = 9)	0%
*ARS605*	ACS…AGGAAACT**TTA**CAAAGCGT	64%	(n = 6)	11%	4%	(n = 6)	1%
	ACS…AGGAAACT**TTA**acAAGCGT	53%	(n = 6)	4%	19%	(n = 6)	2%
	ACS…AGGAAACT**Tcc**CAAAGCGT	35%	(n = 6)	12%	40%	(n = 6)	5%
	ACS…AGGAAACc**cTA**CAAAGCGT	56%	(n = 6)	8%	19%	(n = 6)	2%
	ACS…AGGAAcaT**TTA**CAAAGCGT	64%	(n = 6)	20%	9%	(n = 6)	6%
*ARS319*	ACS…TAGTGGTG**ATT**TTTCTGTA	64%	(n = 12)	13%	6%	(n = 9)	2%
	ACS…TAGTttTG**ATT**TTTCTGTA	66%	(n = 12)	17%	3%	(n = 9)	0%
	ACS…TAGTGGct**ATT**TTTCTGTA	70%	(n = 12)	11%	2%	(n = 9)	1%
	ACS…TAGTGGTG**ggT**TTTCTGTA	63%	(n = 12)	12%	4%	(n = 9)	1%
	ACS…TAGTGGTG**ATg**gTTCTGTA	69%	(n = 12)	14%	8%	(n = 9)	3%
	ACS…TAGTGGTG**ATT**TggCTGTA	65%	(n = 12)	7%	3%	(n = 9)	2%
*ARS317*	ACS…ATTGCGAT**TTA**AAAAATCA	69%	(n = 9)	16%	3%	(n = 9)	3%
	ACS…ATTGCGAT**TTA**ccAAATCA	68%	(n = 9)	12%	4%	(n = 9)	0%
	ACS…ATTGCGAT**Tcc**AAAAATCA	73%	(n = 9)	17%	3%	(n = 9)	1%
	ACS…ATTGCGAc**cTA**AAAAATCA	75%	(n = 9)	15%	2%	(n = 9)	1%
	ACS…ATTGCacT**TTA**AAAAATCA	70%	(n = 9)	5%	2%	(n = 9)	1%
	ACS…ATTaaGAT**TTA**AAAAATCA	71%	(n = 9)	5%	3%	(n = 9)	1%

### Growth media and conditions

*W303* cells (*ade2-1 trp1-1 can1-100 leu2-3,112 his3-11,15 ura3-1)* were routinely grown on rich medium (YPD) at 23°C. Cells transformed with *URA3* integrating fragments or *URA3/CEN4/ARS* mini-chromosomes were selected on Synthetic Complete (SC) medium without uracil. Cells with repressed *URA3* were selected on SC medium supplemented with 1 g/l Fluoro-Orotic Acid (FOA) (Toronto Chemicals).

### Telomere position effect (TPE) assays

Fragments containing *ADH4, URA3, ARS* and telomeric *TG*_*1-3*_ repeats were released by digestion of pUCAIV derivatives with SalI and EcoRI and used to transform *W303* cells. This treatment efficiently integrates the constructs between *ADH4* and the *VIIL* telomere [27]. Telomeric integration was confirmed by PCR and variegated expression of *URA3*[27]. To warrant the loss of un-integrated constructs (these are linear DNAs lacking *CEN* elements), transformants were re-streaked on Sc-ura and SC/FOA plates and then an isolated colony from the SC-ura plate was grown for 20 generations in non-selective (YPD) medium. Serial 1:10 dilutions were prepared and 5 μl aliquots were spotted on SC and SC/FOA plates. The % FOA^R^ for each independent culture was calculated. Each construct was analysed in triplicate (three independent colonies per transformation) in three or more independent transformations. The average values and standard deviation from these experiments were calculated in Microsoft Excel and are shown in Tables 2 and 1. 

**Table 2:** **Silencer activity of *ARSs *cloned in opposite orientations**

**Silencer activity (FOA-resistance assay)**			
	**FOA**^**R**^		**STD**
*no ARS*	36%	(n = 9)	4%
*…URA3-ACS319-B1-tel*	81%	(n = 9)	6%
*…URA3-ACS317-B1-tel*	77%	(n = 9)	5%
*…URA3-ACS305-B1-tel*	78%	(n = 9)	12%
*…URA3-ACS605-B1-tel*	67%	(n = 9)	11%
*…URA3-B1-ACS319-tel*	7%	(n = 9)	7%
*…URA3-B1-ACS317-tel*	21%	(n = 9)	11%
*…URA3-B1-ACS305-tel*	47%	(n = 9)	7%
*…URA3-B1-ACS605-tel*	26%	(n = 9)	8%

### Mini-chromosome stability assay

The cloned and mutated *ARSs* were released from pUCAIV by digestion with BamH1 and sub-cloned in a pUC119 based mini-chromosome [2] containing *URA3* and *CEN4*. Each mini-chromosome was independently transformed in *W303* cells. Three colonies were isolated from SC-ura plates, re-streaked on SC-ura plates and suspended in non-selective (YPD) medium. The cultures were grown for 20 generations in non-selective YPD medium. Serial 1:10 dilutions of the cultures prior and after growth in non-selective medium were prepared and 5 μl aliquots were spotted on SC and SC-ura plates. The *per cent* of *ura+* cells prior and after growth in non-selective medium were used to calculate the mini-chromosome loss per generations as in [22,26]. Each mini-chromosome was analysed in triplicate (three independent colonies per transformation) in three independent transformations. The average values and standard deviation were calculated in Microsoft Excel and are shown in Table 2.

## Abbreviations

W: A/T; Y: C/T; R: A/C; *ARS*: Autonomously Replicating Sequences; *ACS*: *ARS* Consensus Sequence; ORC: Origin Recognition Complex; FOA: 5-fluoro-orotic acid; TPE: Telomere Position Effect.

## Competing interests

The authors declare that they have no competing interests.

## Authors’ contributions

PC-R conceived and participated in the design of the study, cloned and mutagenized the analyzed *ARSs*, carried out the silencing and some of the replicator experiments and drafted the manuscript. SP and KS carried out the replicator assays and prepared figures. KY conceived and participated in the design of the study and wrote the manuscript. All authors have read and approved the manuscript.

## References

[B1] Rusche LN Kirchmaier AL Rine J The establishment, inheritance, and function of silenced chromatin in Saccharomyces cerevisiae Annu Rev Biochem 2003 72 481 516 10.1146/annurev.biochem.72.121801.161547 12676793

[B2] Marahrens Y Stillman B A yeast chromosomal origin of DNA replication defined by multiple functional elements Science 1992 255 5046 817 823 10.1126/science.1536007 1536007

[B3] Rao H Marahrens Y Stillman B Functional conservation of multiple elements in yeast chromosomal replicators Mol Cell Biol 1994 14 11 7643 7651 7935478 10.1128/mcb.14.11.7643-7651.1994 PMC359300

[B4] Rehman MA Wang D Fourel G Gilson E Yankulov K Subtelomeric ACS-containing proto-silencers act as antisilencers in replication factors mutants in Saccharomyces cerevisiae Mol Biol Cell 2009 20 2 631 641 19005221 10.1091/mbc.E08-01-0099 PMC2626567

[B5] Lee DG Bell SP Architecture of the yeast origin recognition complex bound to origins of DNA replication Mol Cell Biol 1997 17 12 7159 7168 9372948 10.1128/mcb.17.12.7159 PMC232573

[B6] Rao H Stillman B The origin recognition complex interacts with a bipartite DNA binding site within yeast replicators Proc Natl Acad Sci USA 1995 92 6 2224 2228 10.1073/pnas.92.6.2224 7892251 PMC42456

[B7] Theis JF Newlon CS The ARS309 chromosomal replicator of Saccharomyces cerevisiae depends on an exceptional ARS consensus sequence Proc Natl Acad Sci USA 1997 94 20 10786 10791 10.1073/pnas.94.20.10786 9380711 PMC23486

[B8] Chang F Theis JF Miller J Nieduszynski CA Newlon CS Weinreich M Analysis of chromosome III replicators reveals an unusual structure for the ARS318 silencer origin and a conserved WTW sequence within the origin recognition complex binding site Mol Cell Biol 2008 28 16 5071 5081 10.1128/MCB.00206-08 18573888 PMC2519699

[B9] Wilmes GM Bell SP The B2 element of the Saccharomyces cerevisiae ARS1 origin of replication requires specific sequences to facilitate pre-RC formation Proc Natl Acad Sci USA 2002 99 1 101 106 10.1073/pnas.012578499 11756674 PMC117521

[B10] Zou L Stillman B Assembly of a complex containing Cdc45p, replication protein A, and Mcm2p at replication origins controlled by S-phase cyclin-dependent kinases and Cdc7p-Dbf4p kinase Mol Cell Biol 2000 20 9 3086 3096 10.1128/MCB.20.9.3086-3096.2000 10757793 PMC85601

[B11] Theis JF Newlon CS Domain B of ARS307 contains two functional elements and contributes to chromosomal replication origin function Mol Cell Biol 1994 14 11 7652 7659 7935479 10.1128/mcb.14.11.7652-7659.1994 PMC359301

[B12] Palacios DeBeer MA Muller U Fox CA Differential DNA affinity specifies roles for the origin recognition complex in budding yeast heterochromatin Genes Dev 2003 17 15 1817 1822 10.1101/gad.1096703 12897051 PMC196224

[B13] Chang F May CD Hoggard T Miller J Fox CA Weinreich M High-resolution analysis of four efficient yeast replication origins reveals new insights into the ORC and putative MCM binding elements Nucleic Acids Res 2011 39 15 6523 6535 10.1093/nar/gkr301 21558171 PMC3159467

[B14] Weinreich M Liang C Chen HH Stillman B Binding of cyclin-dependent kinases to ORC and Cdc6p regulates the chromosome replication cycle Proc Natl Acad Sci USA 2001 98 20 11211 11217 10.1073/pnas.201387198 11572976 PMC58709

[B15] Zou Y Yu Q Bi X Asymmetric positioning of nucleosomes and directional establishment of transcriptionally silent chromatin by Saccharomyces cerevisiae silencers Mol Cell Biol 2006 26 20 7806 7819 10.1128/MCB.01197-06 16908533 PMC1636860

[B16] Zou Y Yu Q Chiu YH Bi X Position effect on the directionality of silencer function in Saccharomyces cerevisiae Genetics 2006 174 1 203 213 10.1534/genetics.106.055525 16783020 PMC1569783

[B17] Bi X Braunstein M Shei GJ Broach JR The yeast HML I silencer defines a heterochromatin domain boundary by directional establishment of silencing Proc Natl Acad Sci USA 1999 96 21 11934 11939 10.1073/pnas.96.21.11934 10518554 PMC18390

[B18] Walmsley RW Chan CS Tye BK Petes TD Unusual DNA sequences associated with the ends of yeast chromosomes Nature 1984 310 5973 157 160 10.1038/310157a0 6377091

[B19] Chan CS Tye BK Organization of DNA sequences and replication origins at yeast telomeres Cell 1983 33 2 563 573 10.1016/0092-8674(83)90437-3 6345000

[B20] Fourel G Lebrun E Gilson E Protosilencers as building blocks for heterochromatin Bioessays 2002 24 9 828 835 10.1002/bies.10139 12210519

[B21] Rehman MA Yankulov K The dual role of autonomously replicating sequences as origins of replication and as silencers Curr Genet 2009 55 4 357 363 10.1007/s00294-009-0265-7 19633981

[B22] Tye BK Minichromosome maintenance as a genetic assay for defects in DNA replication Methods 1999 18 3 329 334 10.1006/meth.1999.0793 10454994

[B23] Yankulov K Dare to challenge the silence? Telomeric gene silencing revisited Nucleus 2011 2 6 513 516 10.4161/nucl.2.6.17710 22064468

[B24] Ottaviani A Gilson E Magdinier F Telomeric position effect: from the yeast paradigm to human pathologies? Biochimie 2008 90 1 93 107 10.1016/j.biochi.2007.07.022 17868970

[B25] Rusche LN Kirchmaier AL Rine J Ordered nucleation and spreading of silenced chromatin in Saccharomyces cerevisiae Mol Biol Cell 2002 13 7 2207 2222 10.1091/mbc.E02-03-0175 12134062 PMC117306

[B26] Kramer DJ Gauthier L Yankulov K Higher-accuracy method for measuring minichromosome stability in Saccharomyces cerevisiae Biotechniques 2002 32 5 1036 1040 12019776

[B27] Gottschling DE Aparicio OM Billington BL Zakian VA Position effect at S. cerevisiae telomeres: reversible repression of Pol II transcription Cell 1990 63 4 751 762 10.1016/0092-8674(90)90141-Z 2225075

[B28] Fourel G Revardel E Koering CE Gilson E Cohabitation of insulators and silencing elements in yeast subtelomeric regions EMBO J 1999 18 9 2522 2537 10.1093/emboj/18.9.2522 10228166 PMC1171334

[B29] Huang RY Kowalski D Multiple DNA elements in ARS305 determine replication origin activity in a yeast chromosome Nucleic Acids Res 1996 24 5 816 823 10.1093/nar/24.5.816 8600446 PMC145715

[B30] Stillman B Origin recognition and the chromosome cycle FEBS Lett 2005 579 4 877 884 10.1016/j.febslet.2004.12.011 15680967

[B31] Weinreich M Palacios DeBeer MA Fox CA The activities of eukaryotic replication origins in chromatin Biochim Biophys Acta 2004 1677 1–3 142 157 15020055 10.1016/j.bbaexp.2003.11.015

[B32] Lipford JR Bell SP Nucleosomes positioned by ORC facilitate the initiation of DNA replication Mol Cell 2001 7 1 21 30 10.1016/S1097-2765(01)00151-4 11172708

[B33] Murrell A Setting up and maintaining differential insulators and boundaries for genomic imprinting Biochem Cell Biol 2011 89 5 469 478 10.1139/o11-043 21936680

